# Laboratory Investigation on Dynamic Complex Modulus of FRPU Composite

**DOI:** 10.3390/ma17246229

**Published:** 2024-12-20

**Authors:** Jarosław Górszczyk, Konrad Malicki, Arkadiusz Kwiecień

**Affiliations:** 1 Faculty of Civil Engineering, Cracow University of Technology, 31-155 Cracow, Poland; kmalicki@pk.edu.pl; 2 FlexAndRobust Systems Ltd., 24 Warszawska Str., 31-155 Cracow, Poland; ak@flexandrobust.com

**Keywords:** viscoelastic material, complex modulus, dynamic mechanical analysis (DMA), storage modulus, loss modulus, glass fibre-reinforced polyurethane, FRP composite, glass fibre

## Abstract

Civil engineering structures are subject to both static and dynamic loadings. This applies especially to buildings in seismic areas as well as bridges, viaducts, and road and railway structures loaded with road or rail traffic. One of the solutions used to repair and strengthen such structures in the event of emergency damage are fibre-reinforced polyurethanes (FRPUs). The article proposes a laboratory method for determining the dynamic complex modulus of FRPU composite tape. The theoretical basis for determining the complex modulus for the tested material is presented. Laboratory tests were carried out using the tensile method for four cyclic loading frequencies and a cyclic load ratio equal to 0.5. Under the assumed test conditions, the material showed a viscoelastic performance with a dominant elastic part (storage modulus). For a frequency of 0.1 Hz, the viscous part (loss modulus) was about 8% of the storage modulus value, while for a frequency of 10 Hz, this value was about 5%. For a loading frequency of 0.1 Hz, the elastic part of the complex modulus was about 1160 MPa, while for a frequency of 10 Hz, it was about 1790 MPa. With the increase in loading frequency, the absolute value of the complex modulus increased.

## 1. Introduction

Civil engineering structures are subject to various loadings. Often, these loads are static. However, in certain conditions, dynamic loads can also occur as main loads. In buildings, this type of load may be caused by earthquakes. In road and railway structures, dynamic forces come mainly from road and rail traffic. As a result of earthquakes or repeated dynamic loadings, partial or complete damage to the structure may occur. In the event of damage to a building or bridge, emergency repairs for strengthening may be needed. In such cases, one of the solutions may be the use of a fibre-reinforced polyurethane (FRPU) composite.

Building materials made of polymers are increasingly used in civil engineering and are subjected to various tests [1]. Liu et al. presented a review on the mechanical properties of pultruded fibre-reinforced polymer (FRP) composites [2]. It was stated that FRP composites are widely used due to their numerous advantages. The main advantages are light weight, corrosion resistance, and high strength. FRP composites can be constructed from many different types of fibres, including carbon, glass, and basalt fibres. FRP structures can be considered as an alternative to conventional concrete or steel structures. However, the long-term performance and degradation of FRP should also be taken into account. Factors such as alkaline/acidic solutions, low/high temperature, UV radiation, and freeze–thaw cycles may negatively affect the parameters of the FRP composite [2]. Righetti et al. studied the effect of storing the glass fibre-reinforced polymer (GFRP) grid in deionized water and a NaCl solution on the mechanical properties of this material [3]. A decrease in the tensile strength and Young’s modulus up to 30.2% and 13.2% was observed. Keller et al. analysed the long-term performance of a glass fibre-reinforced polymer truss bridge [4]. The visual inspection showed various local defects such as crushing, cracks due to the inappropriate storage and lifting of the structure, fibre blooming, and damage due to vandalism. However, the safety and serviceability of the structure have not been affected by these defects.

Kwiecień and Akyildiz presented selected solutions for the protection of reinforced concrete structures with infill walls against earthquakes [5]. The use of polyurethane flexible joints (PUFJs) and FRPU composites was discussed. PUFJs provide a seismic protection between masonry elements and reinforced concrete frames. FRPU composites are used as composite tapes located on the surface of the strengthened structure. Such solutions allow for maintaining the load-bearing capacity and safety of structures subjected to earthquakes. Examples of serious damage to buildings not protected against earthquakes were shown. The results of quasi-static and dynamic tests using harmonic loadings were presented. Wall frames strengthened with the FRPU composite showed a high load-bearing capacity and ductility. Despite the dynamic excitation, the tested building kept its elastic performance. Similar research results were presented by Rousakis et al. [6]. The obtained results confirmed the high efficiency of PUFJs and FRPU composites in terms of reducing seismic loading. Almusallam and Al-Salloum stated that the test results showed that a significant gain in flexural strength can be achieved by bonding the FRP composite to the face of a reinforced concrete element [7]. Nowak-Michta et al. also reported that FRPU composites show a load and deformation capacity as well as durability and water resistance [8].

Gao et al. conducted a study on the mechanical properties and selected applications of GFRP [9]. It was stated that lateral deformation of the GFRP pole showed a linear growth with an increase in load. This indicates that GFRP pole behaviour is linear and elastic. Alshurafa et al. used the finite element method (FEM) for the static and dynamic analysis of a GFRP guyed tower [10]. Gaetani et al. conducted an investigation on the tensile response of GFRP elements through a discrete lattice modelling approach [11]. However, in all these studies and analyses, the GFRP complex modulus was not used.

Dynamic loadings are used in the testing and analysis of various materials and structures used in civil engineering [12,13]. In many tests, material samples are subject to repeated dynamic loading cycles, resulting in the fatigue process. For example, Sołkowski et al. presented the effect of a fatigue test on the mechanical properties of cellular polyurethane mats used in tram and railway tracks [14]. Cyclic hardening was observed, which changed the value of the dynamic bedding modulus during the fatigue process. Zanzinger et al. analysed the fatigue behaviour of a PET-geogrid under cyclic loading [13]. Special clamping was performed using capstan clamps consisting of a mobile half-cylinder. The cyclic load ratio was set to 0.5 and the loading frequency was equal to 3 Hz and 10 Hz. It was found that there is a significant impact of the load frequency on the material lifetime. The higher frequency results in a lower lifetime. This result makes it possible to measure the geogrid against operational loadings.

Laboratory tests are also carried out to determine the viscoelastic parameters of various materials. The analysis of the complex modulus and complex Poisson’s ratio of solid materials were presented by Pritz already in 1998 [15]. It was stated that all real solid materials possess both elastic and damping properties. The complex modulus is a useful tool for characterizing the elastic and viscous properties of material in the frequency domain. The test results show that the storage modulus of polymeric materials increases with increasing frequency. The measured dependences of the complex modulus of polymers are often interpreted by means of viscoelastic models. Both the real and imaginary parts of the dynamic complex modulus are expected to be frequency dependent. The complex modulus is determined by measurement and depends on the type of material and deformation [15]. Choi et al. presented the measurement of viscoelastic constants of a carbon fibre-reinforced (CFR) composite using dynamic mechanical analysis (DMA) and the digital image correlation (DIC) method [16]. A three-point bending test with a load frequency of 1 Hz was used. It was reported that the Poisson’s ratio of polymeric materials, which affects the value of the complex modulus, shows a viscoelastic dependence on time, temperature, and deformation. At a temperature of 30 °C, the storage modulus value (describing the elastic component of the complex modulus) was approximately 1600 MPa and the loss modulus value (describing the viscous component of the complex modulus) was approximately 300 MPa. Viscoelastic parameters are also determined for composites such as bituminous mixtures [17,18]. Gudmarsson et al. presented the test results of the complex modulus and complex Poisson’s ratio from the dynamic testing of asphalt concrete [18]. Specimens have been tested through modal testing using impact hammer and cyclic tension–compression measurements. Using frequency response functions (FRFs), it was possible to determine the master curves of the asphalt mixture. These curves describe the viscoelastic material parameters as a function of frequency and temperature.

In this work, two research goals were defined. The first goal was to develop a laboratory stand and a research method for testing the complex modulus of the FRPU composite tape. The second aim of the research was to determine the influence of the load frequency on the complex modulus of the FRPU composite using the proposed method.

## 2. Materials and Methods

### 2.1. Tested Material

**Figure 1:**
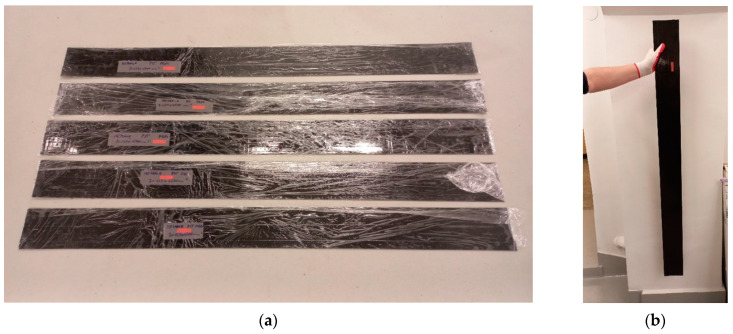
View of specimens before testing: (**a**) the series of specimens delivered to the laboratory; (**b**) the specimen immediately before clamping in the testing machine.

Tests were carried out on a series of five FRPU composite specimens. The FRPU composite was constructed from a glass fibre grid embedded in a PS type polyurethane matrix. The PS designation is the trade name of the product of FlexAndRobust Systems Ltd. (Cracow, Poland) [19]. This polyurethane is a solvent-free elastic two-component material. The FRPU composite is used to strengthen civil engineering structures [5]. Specimens with the following dimensions were used: width of 127 mm, length of 1500 mm, and thickness of 3 mm. The series of specimens prepared for testing is shown in Figure 1.

The longest dimension of the specimen coincided with the warp direction of the glass grid. The tested composite is used as a reinforcing tape working in the longitudinal direction. Therefore, this direction was tested. All specimens used in the test were in good technical condition with no visible surface damage. Selected parameters of the tested material are shown in Table 1 [19,20,21].

### 2.2. Test Methods and Parameters

The complex modulus $E*\left(\omega \right)$, as a basic parameter characterizing the viscoelastic behaviour of the material, is defined by Equation (1):

(1) $$E*\left(\omega \right)=\frac{\sigma_{0}}{\varepsilon_{0}}e^{i\Theta }=\left|E*\right|e^{i\Theta }=E^{\prime }\left(\omega \right)+iE^{\prime \prime }\left(\omega \right),$$

where $\sigma_{0}$ is the stress amplitude, $\varepsilon_{0}$ is the corresponding steady-state strain amplitude, *ω* is the angular frequency, $i=\sqrt{-1}$ is the imaginary unit, Θ is the phase angle, $\left|E*\right|$ is the absolute value of the complex modulus, $E^{\prime }\left(\omega \right)$ is the real part (the storage modulus), and $E^{\prime \prime }\left(\omega \right)$ is the imaginary part (the loss modulus). The storage modulus represents the energy stored in the elastic structure of the material. The loss modulus represents the viscous part or the amount of energy dissipated in the material.

The storage modulus and the loss modulus are often determined using DMA experiments. In this type of experiment, a material is subjected to a sinusoidal stress (controlled force method) or strain (controlled displacement method), and the material’s steady-state response to this imposed strain or stress is measured. Since there are no appropriate standards for the dynamic testing of this type of product, it was necessary to develop a testing procedure in order to perform the DMA of composite. The laboratory test method was developed based on standards for similar materials, i.e., EN 12697-26 [23] and EN ISO 10319 [24].

Before starting the tests, the specimens were conditioned on a flat surface at the test temperature for a minimum of 4 h. The specimens were then mounted in the roller grips of the testing machine.

Prior to the testing load, a preload was performed to eliminate any slack in the specimen mounting system. Then, a time-dependent sinusoidal axial load was generated with a load ratio of 0.5 (R = 0.5) and a minimum value of 1.0 kN. The controlled force method was used. Test parameters were established based on our own preliminary tests and the literature review, e.g., [13,23] and ([24], p. 10). The same load cycle parameters were used for all four load frequencies: 0.1, 1.0, 5.0, and 10 Hz. The test parameters are listed in Table 2.

The test parameters were the imposed tensile force and its corresponding measured displacement. The nominal stresses in the specimen were determined as the quotient of the tensile force and the initial cross-sectional area of the tested specimen. The strains were calculated for an initial gauge length of 80 mm, which was marked on the specimen. The tests were carried out at a room temperature of +21 ± 1 °C. The complex modulus was calculated after the target cyclic force/stress values had stabilized. This necessary phase of stabilization of the test conditions ranged from 2 cycles for a frequency of 0.1 Hz to approx. 100 cycles for a frequency of 10 Hz.

For the above-mentioned test conditions, the complex modulus E* as a complex number was calculated using the polar form according to the equations below (2)–(5):

E* = |E*|*(cos(Φ) + i∙sin(Φ))* (2)

*Φ* = ∆*t∙2π∙f* (3)

|E*| = *Fa ∙l∙(A*∙∆*l)^−1^* (4)

*E*′ = |E*|*cos(Φ)*,      *E*″ = |E*|*sin(Φ)* (5)

where $\left|E*\right|$ is the absolute value of the complex modulus, $i=\sqrt{-1}$ is the imaginary unit, ∆*t* is the time lag between stress and strain signals, *Φ* is the phase angle, *f* is the ordinary frequency, *Fa* is the load amplitude, *A* is the cross-sectional area of the specimen, *l* is the nominal gauge length, ∆*l* is the change amplitude in the gauge length, $E^{\prime }$ is the real part (the storage modulus), and $E^{\prime \prime }$ is the imaginary part (the loss modulus).

### 2.3. Laboratory Set-Up

**Figure 2:**
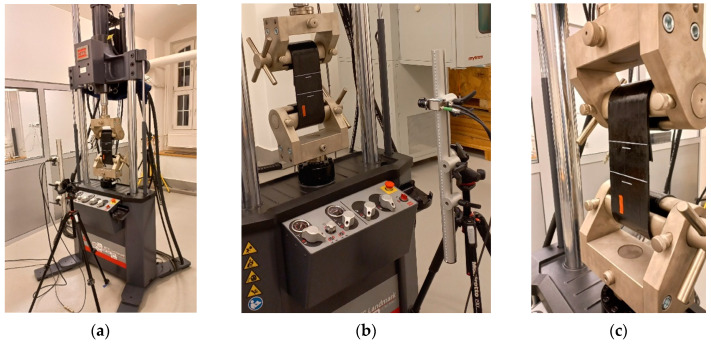
Testing stand: (**a**) general view; (**b**) view of the FRPU specimen and the RTSS measurement system camera; (**c**) the specimen fix on roller grips with visible white marking for the videoextensometer gauge.

The implementation of the tests for the assumptions described above required the preparation of a special laboratory stand. The stand enabled the generation of cyclic tensile loads on the sample so that the sample did not slide out or break in the grips. The use of typical clamping jaws is problematic here, because they cause the composite matrix to be crushed at the edge of the jaw and this distorts the test results. In order not to damage the composite specimen while clamping it in the grips, it was necessary to use specially prepared double roller grips. The design of the roller grips effectively protected the specimen from slipping out during dynamic cyclic tests. The appropriate radius of the roller curvature protected the composite tape from breaking on the roller surfaces (Figure 2c). The roller grips were installed in the dynamic testing machine. The MTS Landmark Servohydraulic Test System with a force sensor accuracy class of 0.5 was used in the tests. The laboratory stand was expanded with the Dantec Dynamics RTSS (real time strain sensor) videoextensometer, enabling non-contact measurements of displacements and strains. The precision class for the RTSS system equals 1.0 [25]. White lines for the measurement base were permanently marked on the surface of the specimen. The length of the measurement gauge was selected based on standard recommendations [21]. The laboratory stand with the specimen prepared for testing is shown in Figure 2.

## 3. Results and Discussion

### 3.1. Analysis of the Test Results for a Cyclic Loading Frequency of 5 Hz

**Figure 3:**
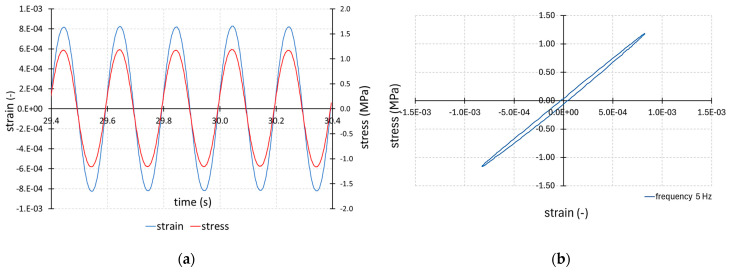
Stress and strain signal of the selected specimen for frequency 5 Hz: (**a**) in the time domain; (**b**) the hysteresis loop.

This section shows a full analysis of the results obtained for a selected load frequency of 5 Hz. The stress and strain obtained in the tests in the time domain as excitation and response signals for the selected specimen and for the frequency of 5 Hz are shown in Figure 3a. For the stress and strain cycles, the average values were zeroed. The relationship between the imposed stress and the obtained strain for the selected moment of time from this range can also be presented in the form of a hysteresis loop. Such an approach to the relationship between stress and strain is shown in Figure 3b. From the shape of the hysteresis loop, it is possible to determine the energy loss in the cycle. This is the visible area of the hysteresis loop. The dissipation of energy in the cycle is caused by the viscous properties of the matrix material.

The strain signal obtained in the tests showed the correct change in time in the form of a harmonic waveform (Figure 3a). In comparison to the imposed stress signal, the strain as a response signal showed a lag in the time domain. The complete composite specimen results from all tests with a cyclic loading frequency of 5 Hz are shown in Table 3.

The obtained results for a cyclic loading frequency of 5 Hz confirmed that the FRPU composite behaves as a viscoelastic material. All results were statistically verified for the possibility of outlier values. Based on the Q-Dixon’s statistical test, one of the test results for the frequency of 5 Hz was found as the outlier result at the significance level of α = 0.05. In a further analysis, this result (FRPU-4) was rejected. This was the only situation and did not occur for other frequencies. The occurrence of a single outlier result could have been caused by an error in the measuring system. The absolute value of the complex modulus was 1534 ± 97 MPa. The phase angle was 0.07 ± 0.0066 rad. The obtained results are similar to those presented in the literature [16]. A relatively small dispersion of results was obtained. The maximum coefficient of variation was 9.5% (for the phase angle).

### 3.2. Analysis of the Effect of Cyclic Load Frequency on the Value of the Complex Modulus and Phase Angle

**Figure 4:**
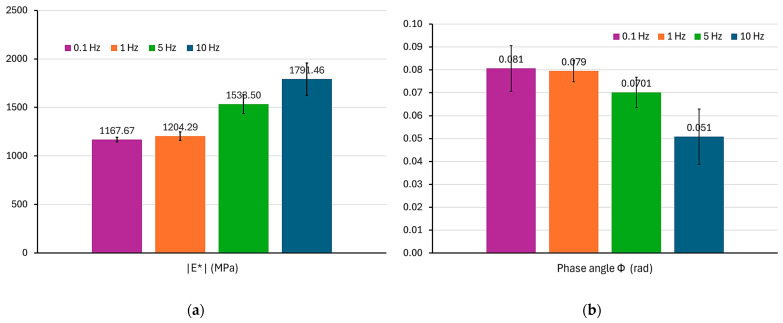
Mean values and standard deviations: (**a**) absolute value of the complex modulus; (**b**) phase angle (argument) of the complex modulus.

An identical analysis to the one described in Section 3.1 was performed for all frequencies. The evaluation of the results began with the statistical analysis. No outlier results were found. The test results of the dynamic complex modulus for all frequencies in the form of mean values are summarized in Table 4. The storage modulus E′ and the loss modulus E″ were calculated from the mean values of the absolute complex modulus and the mean phase angle. The statistical dispersion of results is shown in summary form in Figure 4.

In order to check the statistical significance of differences between the mean values of the parameters presented in Figure 4, an analysis of variance (ANOVA) was performed. First, the assumptions of the analysis were checked. The normality of the variable distributions was confirmed at the significance level of α = 0.05 using the Shapiro–Wilk statistical test. Using Levene’s statistical test, the assumption of homogeneity of variance was confirmed at the significance level of α = 0.05. Using the Tukey post hoc test, the occurrence of statistically significant differences between the selected mean values was confirmed. The results are presented in Table 5. In this table, the symbol [x] denotes the occurrence of statistically significant differences between the mean values at the significance level of α = 0.05. The symbol [-] denotes the absence of such differences.

The mean values of the absolute value of the complex modulus for the frequencies of 5 Hz and 10 Hz showed statistically significant differences with the mean values for all other frequencies. No significant difference was found between the mean values for the frequencies of 0.1 Hz and 1 Hz. The mean values of the phase angle for the frequency of 10 Hz showed statistically significant differences with the mean values for the frequencies of 0.1 Hz and 1 Hz. No statistically significant differences were found between the mean values of the phase angle for the other frequencies.

An interesting trend was observed in Figure 4 regarding the dispersion of the complex modulus and phase angle results. The largest dispersion of results (standard deviation) was observed for the frequency of 10 Hz. Apart from the frequency of 0.1 Hz, generally, with the decrease in the frequency, a smaller dispersion of results was observed. The increase in the dispersion of results with the increase in the frequency could be caused by the more intensive dynamics of the test system.

**Figure 5:**
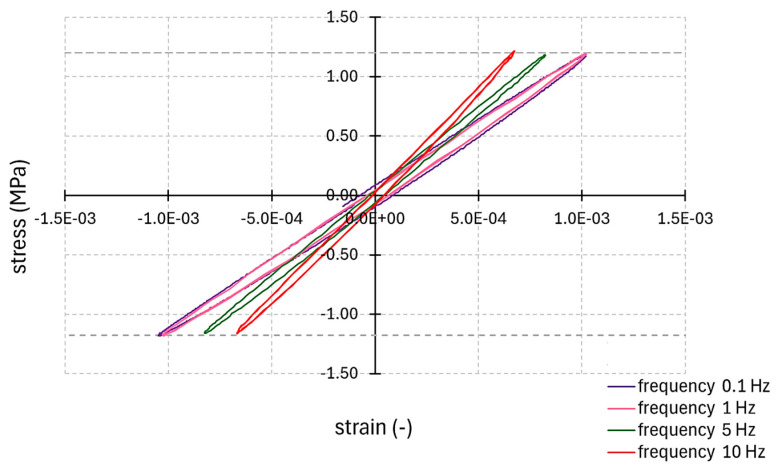
Selected hysteresis loops for all cyclic load frequencies.

In the next step, the relationships between the imposed stress and the obtained strain in the form of a hysteresis loop for all frequencies were analysed. The hysteresis loops for the selected samples are shown in Figure 5.

The obtained hysteresis loops showed a correct trend in material performance due to the change in cyclic loading frequency (Figure 5). With the increase in frequency, the hysteresis loops rotated in the vertical direction (the absolute value of the dynamic modulus increases). With the increase in frequency at the same imposed stress value, a smaller strain value occurred. For lower frequencies, i.e., 0.1 Hz and 1 Hz, the effect of frequency was small. The loops almost overlapped. The effect of frequency was more visible at higher dynamic loading frequencies. This is consistent with the results shown in Table 4. A similar trend was also observed in the change in the area inside the hysteresis loop. For low frequencies, the loop area was larger; for higher frequencies, the width of the loop area was smaller. This indicates a decrease in the viscous component in the material’s work with the increase in the loading frequency and confirms the viscoelastic nature of the material’s performance.

**Figure 6:**
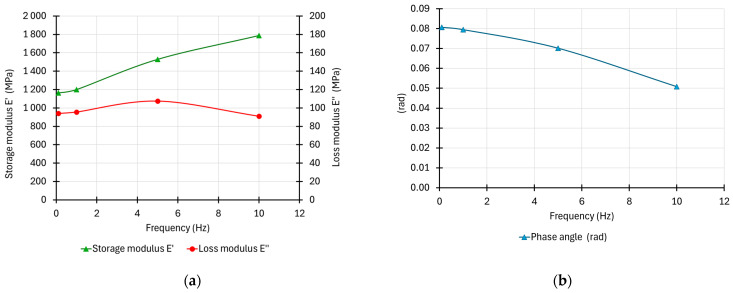
(**a**) Storage and loss modulus plots in the frequency domain—mean values; (**b**) phase angle plot in the frequency domain—mean values.

In the next step, the storage and loss modulus plots as well as the phase angle plot in the frequency domain were analysed. The analysed curves are shown in Figure 6.

In Figure 6a, the storage modulus value increases with the increasing frequency in the entire analysed area. In the central part of the plot, the modulus increase is the largest. The graph flattens out at the edges of the analysed area. The elastic part (storage modulus) of the complex modulus assumes values from 1150 MPa for a frequency of 0.1 Hz to 1780 MPa for a frequency of 10 Hz. Therefore, comparing the quasistatic load (0.1 Hz) with the load with a frequency of 10 Hz, there is a significant increase in the elastic modulus value by about 50%. The loss modulus value showed a curvilinear variability in the analysed range, close to a parabola. A local maximum of the curve was observed for a load frequency close to 5 Hz. Such a graph is consistent with the literature [16]. For a frequency of 0.1 Hz, the viscous part constitutes about 8% of the storage modulus value. For a frequency of 5 Hz, it is about 7%. For a frequency of 10 Hz, the viscous part constitutes only about 5% of the storage modulus value. This confirms the increase in the storage modulus value with an increasing load frequency.

In Figure 6b, a constant decrease in the phase angle value can be observed with the increase in the cyclic loading frequency. For the frequency of 0.1 Hz, the angle was 0.08 rad, while for the frequency of 10 Hz, it dropped to 0.05 rad. This variability also confirms the decrease in the viscous component in the work of the material with the increase in frequency. A small value of the angle indicates a more elastic performance of the tested composite material.

**Figure 7:**
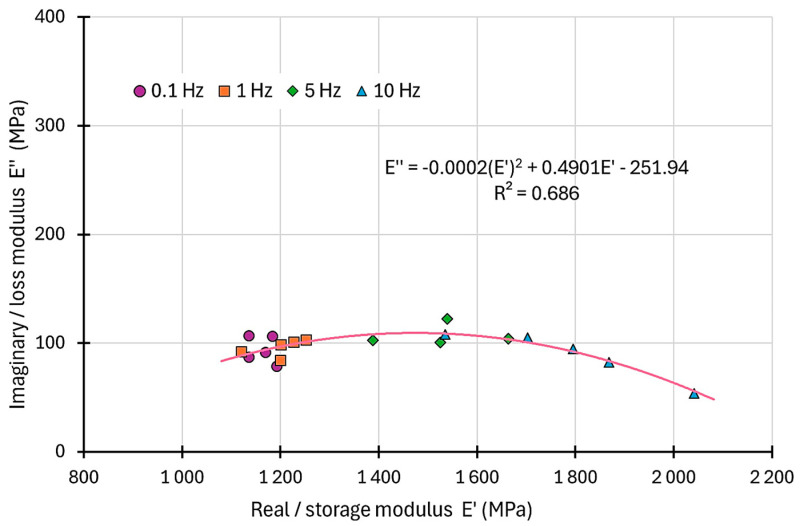
Dynamic complex modulus results of the complex plane for all samples and all frequencies.

Finally, the complex modulus results of the complex plane were analysed. The analysed results are shown in Figure 7.

The presentation of the results from the complex plane allows for observing the global trend of their distribution. The results for low frequencies are located on the left side of the graph, while the higher frequencies results are located on the right side. On this basis, it is possible to determine the distribution function. A second-degree polynomial function was used. A fairly good fit of the results was obtained. The determination coefficient R^2^ was approximately 0.68. The presented results are of significant importance from the point of view of recognizing the dynamic properties of modern composite materials. The obtained results from the laboratory tests can be used to select and calibrate viscoelastic material models. The selection of an appropriate material model for the FRPU composite allows for calculations (e.g., using FEM) for structures strengthened with FRPU composites subjected to dynamic/time-dependent loading. However, the FRPU composite complex modulus depends on many factors, such as temperature, load characteristics, and environmental influences. Moreover, the long-term performance of the tested material is important. Therefore, further and broader research and analysis of the dynamic performance of FRPU composites are needed.

## 4. Conclusions

The results of the conducted research allow the formulation of the following conclusions:

- In the applied dynamic testing method, no slippage of specimens or damage to the composite matrix surface was observed.
- For the assumed dynamic loading conditions, the tested material showed a viscoelastic character with a dominant elastic part.
- With the increase in the frequency of cyclic loading, the absolute value of the complex modulus increased. In this case, an increase in the real part of the modulus describing the elastic properties of the composite was observed. For a loading frequency of 1 Hz, the real part of the complex modulus was about 1200 MPa, while for a frequency of 10 Hz, it was about 1790 MPa.
- The loss modulus value showed a curvilinear variability in the analysed range, close to a parabola. A local maximum of the curve was observed for a load frequency close to 5 Hz.
- The complex modulus of the FRPU composite also depends on other factors not examined in this article. Therefore, further research and analysis of the dynamic performance of the FRPU composite are needed.

## Figures and Tables

**Table 1:** Parameters of the tested composite.

Composite Component Parameter	Type/Value
matrix material	polyurethane PS
matrix density	1450 kg/m^3^
matrix static Poisson ratio	0.48
grid material	glass fibres
grid aperture	15 × 15 mm^2^
composite tensile strength [22]	29.6 MPa
composite static modulus [22]	1230 MPa

**Table 2:** Test plan and parameters.

Test	Load Frequency (Hz)	Number of Load Cycles	Temperature (°C)
01	0.1	3	21 ± 1
02	1.0	20	21 ± 1
03	5.0	80	21 ± 1
04	10.0	110	21 ± 1

**Table 3:** Test results of dynamic complex modulus with a cyclic loading frequency of 5 Hz.

Specimen	Absolute Value of the Complex Modulus	Phase Angle	Real Part of the Complex Modulus (Storage Modulus)	Imaginary Part of the Complex Modulus (Loss Modulus)	Exponential form of the Dynamic Complex Modulus
ID	|E*| (MPa)	Φ (rad)	E′ (MPa)	E″ (MPa)	E* (MPa)
FRPU-1	1667.2	0.0622	1663.9	103.6	1667·e^0.062i^
FRPU-2	1392.9	0.0735	1389.1	102.3	1393·e^0.074i^
FRPU-3	1544.5	0.0792	1539.7	122.2	1545·e^0.079j^
FRPU-4	1769.9	0.0914	1762.5	161.5	1770·e^0.091i^
FRPU-5	1529.4	0.0675	1526.1	100.4	1529·e^0.068i^

**Table 4:** Calculated test results of dynamic complex modulus for all frequencies (mean values).

Loading Frequency (Hz)	Exponential form of the Dynamic Complex Modulus E* (MPa)	General form of the Dynamic Complex Modulus E* (MPa)
0.1	1168·e^0.081i^	1164 + 94∙i
1.0	1204·e^0.079i^	1200 + 96∙i
5.0	1533∙e^0.070i^	1530 + 107∙i
10.0	1791∙e^0.051i^	1789 + 91∙i

**Table 5:** ANOVA results for absolute value of the complex modulus and phase angle.

Frequency (Hz)	For Absolute Value of the Modulus				For Phase Angle			
	0.1	1	5	10	0.1	1	5	10
0.1		-	x	x		-	-	x
1	-		x	x	-		-	x
5	x	x		x	-	-		-
10	x	x	x		x	x	-	

## Data Availability

The original contributions presented in the study are included in the article; further inquiries can be directed to the corresponding author.
